# Regulation of vascular remodeling by immune microenvironment after the establishment of autologous arteriovenous fistula in ESRD patients

**DOI:** 10.3389/fimmu.2024.1365422

**Published:** 2024-05-14

**Authors:** Yifei Zhang, Xianglei Kong, Liming Liang, Dongmei Xu

**Affiliations:** Department of Nephrology, The First Affiliated Hospital of Shandong First Medical University & Shandong Provincial Qianfoshan Hospital, Shandong Institute of Nephrology, Jinan, Shandong, China

**Keywords:** arteriovenous fistula, immune microenvironment, inflammation, vascular remodeling, chronic kidney disease

## Abstract

Autogenous arteriovenous fistula (AVF) is the preferred dialysis access for receiving hemodialysis treatment in end-stage renal disease patients. After AVF is established, vascular remodeling occurs in order to adapt to hemodynamic changes. Uremia toxins, surgical injury, blood flow changes and other factors can induce inflammatory response, immune microenvironment changes, and play an important role in the maintenance of AVF vascular remodeling. This process involves the infiltration of pro-inflammatory and anti-inflammatory immune cells and the secretion of cytokines. Pro-inflammatory and anti-inflammatory immune cells include neutrophil (NEUT), dendritic cell (DC), T lymphocyte, macrophage (Mφ), etc. This article reviews the latest research progress and focuses on the role of immune microenvironment changes in vascular remodeling of AVF, in order to provide a new theoretical basis for the prevention and treatment of AVF failure.

## Introduction

Chronic kidney disease (CKD) is a global public health problem. The prevalence of CKD in Chinese adults was 8.2% in the analysis of the results of the Sixth Chronic Disease and Risk Factor Surveillance in China (1), while the prevalence of dialysis in China was 419 cases per million population (2). The three main types of vascular access for hemodialysis (HD) include autogenous arteriovenous fistula (AVF), graft arteriovenous fistula (AVG), and central venous catheter (CVC). AVF refers to artificial formation of a straight line between the proximal artery and the superficial vein at the subcutaneous distance during surgery. AVF has the advantages of lower medical cost, fewer complications, lower infection rate, lower mortality rate and higher patency rate compared with AVG or CVC (3). However, immaturity of newly formed AVF and dysfunction of established AVF are two common clinical challenges (4). The National Institutes of Health reported that 60% of AVF failed to mature after surgery and could not be successfully used for dialysis treatment (5). The hemodialysis arteriovenous fistula maturity (HFM) study showed that the rate of AVF dysfunction at 1 year was 24% (4). The results of our center showed that the 1-year AVF dysfunction rate was 14.4% (6).

The maturation of AVF is the process that makes AVF suitable for providing prescription dialysis. After the construction of AVF, the veins are affected by injury factors such as inflammation and oxidative stress, and exposed to high flow, high shear stress (WSS), high pressure and oxygen-rich arterial blood environment, which requires adaptive remodeling of AVF to mature, that is, the venous cavity dilation and wall thickening are the main characteristics, resulting in venous arterialization changes. Finally, adaptive vascular remodeling related to the acquisition of arteriovenous dual features was formed (7). The immaturity of AVF means that it cannot be used successfully for HD within 6 months after its creation despite surgical intervention. AVF dysfunction is a multifactorial process in which upstream and downstream events work together to lead to lumen stenosis and even secondary thrombosis due to neointimal hyperplasia (NIH). The upstream event refers to the injury of vascular endothelial cell (EC) and vascular smooth muscle cell (VSMC) caused by surgical trauma, hemodynamic shear stress, and uremic toxins. Downstream events refer to responses to vascular damage, including oxidative stress, inflammation, migration and proliferation of fibroblast, SMC, and myofibroblast (8). Activation of EC can increase the expression of growth factors, promote the migration and proliferation of VSMC from the medium to the intima and make it quickly form a new endometrium layer (9, 10). And EC injury, in turn, promotes the release of inflammatory mediators and the recruitment of inflammatory cells, thereby activating smooth muscle cells and fibroblasts and promoting the fibrosis process (11). Fibrosis is an outcome of the dysregulated tissue repair process in response to various types of tissue injury (12). And the degree of fibrosis after AVF surgery is related to the immaturity of AVF. AVF fibrosis can be attributed to excessive extracellular matrix (ECM) synthesis by activated VSMAs and myofibroblasts and/or insufficient ECM degradation by matrix metalloproteinases (MMP). Excessive fibrosis may reduce vessel wall compliance, and AVF patients with intermediate fibrosis have less increase in venous diameter during reconstruction (13). When the vessel wall loses compliance due to excessive ECM deposition, the NIH becomes obstructive (possibly less compressible under high blood flow) and stenotic.

Therefore, fibrotic remodeling and NIH, which in combination significantly increase the risk of AVF failure (14). Our previous study have shown that in the inferior vena cava of adenine-induced chronic kidney disease rats with aortocaval fistulas, in addition to the disordered EC and SMC, inflammatory cells such as macrophage (Mφ) are also present in the major cellular components of NIH (15). In conclusion, inflammation plays an important role in the vascular remodeling process of AVF.

The establishment of AVF can trigger the body’s immune response, including chronic infiltration of immune cells and secretion of inflammatory cytokines (16). The balance between pro-inflammatory and anti-inflammatory responses is necessary for vascular remodeling of AVF, and excessive or insufficient inflammation can lead to AVF failure (16). Since the infiltration of immune cells plays a key role in the vascular remodeling of AVF, it is of great significance to study the changes and roles of immune cells in the vascular remodeling of AVF. In the past, T lymphocyte and Mφ were considered to be key molecules in chronic inflammation of vascular diseases such as atherosclerosis and AVF failure (17). However, recent evidence strongly suggests that neutrophil (NEUT) and dendritic cell (DC) are also involved in the development of vascular disease, such as AVF failure (16, 18). T lymphocyte, Mφ, NEUT, DC and other immune cells participate in the vascular remodeling process of AVF, and the inflammatory response mediated by the above inflammatory cells is the core of the vascular remodeling process (19). This article reviews the role of immune microenvironment in vascular remodeling of AVF.

## Immune cells are involved in vascular remodeling of AVF

Immaturity of AVF refers to the lack of outward remodeling that, despite radiologic or surgical intervention (i.e., endovascular or open procedure management), cannot be successfully used for dialysis after its creation (20). AVF dysfunction is a very general term, and because it does not specifically address the cause of dysfunction, it has been replaced by three terms: thrombotic flow-related dysfunction, non-thrombotic flow-related dysfunction, and infectious dysfunction. Thrombotic flow-related dysfunction is associated with the risk of thrombosis or specific complications that can reduce blood flow to AVF, threaten prescribed dialysis lumen flow and/or cause clinical signs and symptoms such as stenosis or thrombosis. In contrast, non-thrombotic flow-related dysfunction may or may not threaten blood flow or patency, but is associated with clinical signs and symptoms, such as aneurysms and steal syndrome. The above three terms distinguish AVF dysfunction due to stenosis or thrombosis from dysfunction due to other causes (21). Therefore, the dysfunction discussed in this paper refers to thrombotic flow-related dysfunction associated with vascular remodeling.

In the early stages after AVF establishment, the vein is exposed to a high flow, high shear stress, high pressure, and oxygen-rich arterial environment, leading to “maturation” of both the arterial inflow limb and venous outflow limb. Adaptation of the vein to the increased flow and shear stress of the arterial environment requires dilation by outward remodeling of the venous wall (Poiseuille’s law), whereas increases in pressure and tensile stress result in wall thickening (Laplace’s law) (22). WSS is significantly elevated, causing the initial venous cavity to dilate through mechanical stretching, and activating EC to produce large amounts of nitric oxide (NO), stimulating MMP-2, 9 (23), triggering ECM degradation, resulting in continuous dilation of the lumen (24). However, wall thickening is an adaptation of blood vessel walls to pressure, involving the thickening of all vascular layers through ECM deposition and cell proliferation and migration (25). Thus, AVF maturation is an adaptive vascular remodeling associated with the acquisition of dual arteriovenous characteristics. In the later period after AVF establishment, during maintenance HD therapy, repeated treatment placed the region in a state of turbulence, low flow, low wall shear stress, and increased transwall pressure. The normal physiological mechanism of AVF is not clear, but the intima thickening of AVF can maintain sufficient pressure (26).

AVF failure occurs through two different mechanisms: early failure is secondary to lack of outward remodeling and inability to support HD, i.e. AVF immaturity; Late failure is due to uncontrolled pathological process of intimal thickening, uncontrolled NIH, negative or introversion remodeling leading to previously normal intimal thickening, lumen narrowing, and reduced patency rate (17). The formation of NIH can lead to secondary thrombosis and aggravate AVF stenosis (27). The histological features of NIH is characterized by a large number of constricted SMC, myofibroblast, fibroblast, and Mφ, ultimately narrowing the venous outflow tract, and resulting in reduced blood flow (28). However, both NIH and atherosclerosis share partially similar histopathological features: the observed intimal hyperplasia is almost the same, and both are characterized by smooth muscle cell migration and proliferation and ECM deposition (29, 30). Therefore, it can be speculated that there may be chronic inflammation in the NIH of AVF.

The inflammatory response of AVF can be divided into two parts: one is surgical trauma stoma and local hypoxia, which causes local inflammatory response by activating molecular signal transduction such as hypoxia-inducible factor (HIF). Secondly, the presence of uremia in CKD patients promotes oxidative stress, leading to phagocyte activation, oxygen free radical release, lipid peroxidation, and ultimately an increase in systemic inflammatory response (28). Early and late inflammation after AVF establishment plays an important role in the regulation of vascular remodeling. Damage to vascular EC or SMC can induce local immune cell migration (28), as well as damage associated with molecular patterns (DAMP) secretion, which further activates the immune system, recruitment of immune cells, and causes inflammation (31). Thus, inflammation during vascular remodeling in AVF involves a coordinated interaction between blood vessel walls and circulating immune cells (32). In the AVF mouse model, the experimental group lacked radio-protective 105 protein (RP105), also known as CD180- a cell surface protein of various inflammatory cells that has been shown to impair Toll-like receptor (TLR)-4 signaling, thereby reducing outward venous remodeling (33). In a balloon induced rat model of carotid artery injury, NIH was increased in nude mice lacking T lymphocyte, while adoptive transfer of CD4^+^and CD8^+^ lymphocytes in mice after carotid artery injury was associated with decreased NIH (34). Studies in rodent models lacking macrophage colony-stimulating factors found that the Mφ of experimental mice were reduced, and therefore their outward remodeling was attenuated compared to wild-type mice (32). Conversely, adoptive transfer of regulatory T lymphocyte (Treg) has been shown to reduce the incidence of myocardial hypertrophy and fibrosis, as well as the infiltration and remodeling of immune cells (35). In the MISRA’s laboratory, systematic Mφ depletion of a mouse model of AVF with sodium clodronate resulted in a decrease in the number of circulating Mφ and an improvement in AVF stenosis (28, 36). This contradiction may be related to the different roles of T lymphocyte and Mφ in vascular remodeling of AVF (17, 37). Welt and Edelman discovered in 2000 that NEUT played a role in the NIH in balloon-injured arteries of the rabbit model (38). While, Takahashi and Lee discovered in 2003 that DC play a role in the NIH after rat carotid balloon injury (16, 39). Studies have shown that elevated levels of C-reactive protein (CRP), a marked of the systemic inflammatory response produced by the liver in response to pro-inflammatory cytokines, increase the risk of AVF failure, and inhibiting the inflammatory response with prednisolone can promote venous outward remodeling and increase AVF patency in a murine AVF model (40). These findings suggest that appropriate local wall inflammation is positively correlated with AVF vascular remodeling, while systemic inflammation is negatively correlated with AVF vascular remodeling (41). These results suggest that local inflammation driven by immune cells is a necessary condition for vascular remodeling in AVF, and the balance of inflammatory and anti-inflammatory responses is related to the type and number of immune cells infiltrating the AVF site (16). Therefore, immune cells such as T lymphocyte, Mφ, NEUT, and DC play different roles in AVF vascular remodeling (17).

## Different types of immune cells are involved in vascular remodeling of AVF

### Macrophages: play a dual role through polarization

Mφ, DC and monocyte constitute the mononuclear phagocytic system, which is the regulator of tissue homeostasis, growth, development and regeneration (42). The monocyte-to-lymphocyte ratio (MLR) is defined as the absolute number of monocytes divided by the absolute number of lymphocytes. Several studies have found a positive correlation between MLR and AVF immaturity (43). The release of macrophage migration inhibitor factor (MIF) up-regulates tumor necrosis factor (TNF) -α, interleukin (IL) -6, IL-8 and MCP-1 and other inflammatory cytokines, causing the migration and proliferation of inflammatory cells and promoting NIH (19). MCP-1 is involved in the infiltration and activation of monocyte/Mφ, and the intermediate early response gene X-1 (IEX-1) regulates MCP-1, IEX-1 knockout mice reduce MCP-1 and NIH (44), directly blocking MCP-1 inhibition of NIH in different animal models (45). Mφ produce MMP and reactive oxygen species (ROS). MMP can damage elastin fibers such as the inner elastic lamina and promote the dilation of blood vessel walls in a pig model of AVF stenosis (46). However, MMP also promotes the deposition of ECM in NIH and leads to subsequent thrombosis in NIH by breaking down proteins in the ECM (47). However, ROS can alter vascular reactivity, recruit more pro-inflammatory Mφ, and promote vascular remodeling in conjunction with MMP (48). In a mouse model of AVF, injection of sodium clodronate promote Mφ depletion and decreased the proliferation of VSMC activity during venous remodeling (39).

Mφ is a highly plastic type of cell. In a specific tissue with a specific phenotype and function, the process by which Mφ responds to the stimuli from the immune microenvironment is called polarization (49). Mφ is typically found in two distinct subsets: classically activated or M1Mφ, which is pro-inflammatory and polarized by the secretion of IFN-γ by Th1 cells; M2Mφ has anti-inflammatory and immunomodulatory effects and is polarized by IL-4 secreted by Th2 cells and IL-10 and TGF-β secreted by Treg cell (50). Studies have shown that inflammation caused by M1-like Mφ is associated with vein graft failure, participating in the formation of new blood vessels and disrupting the integrity of blood vessels. M2-like Mφ aggregation is associated with SMC proliferation and vascular wall thickening, promoting vascular stability and maturation (42, 51).

#### Classically activated macrophages

M1Mφ produces pro-inflammatory cytokines such as IL-6, IL-12, IL-23, interferon (IFN)-γ and TNF-α and inducible nitric oxide synthase (iNOS). Unlike circulating inflammatory cells, which contribute to systemic inflammation, M1-like Mφ acts within tissues, promoting local inflammation (42). M1-like Mφ produces iNOS, which can reduce vascular remodeling and NIH. Therefore, M1-like Mφ can inhibit AVF maturation and promote AVF function maintenance (17).

#### Vicariously activated macrophages

M2Mφ produces IL-10, transforming growth factor (TGF) -β, arginase (Arg)-1, and other anti-inflammatory cytokines, which have anti-inflammatory and tissue remodeling functions. The appearance of M2-like Mφ is a characteristic of the inflammation resolution period and a necessary condition for vascular graft re-endothelialization, which can promote vascular graft remodeling and facilitate wound healing and tissue reconstruction (52). Studies about the AVF in wild-type C57BL/6J and CD44 knockout mice have shown that CD44 regulates venous wall thickening during AVF maturation by promoting the accumulation of M2-like Mφ in the adaptive venous wall (53). IL-10 and TGF-β play an important role in maintaining vascular integrity: IL-10 and TGF-β produced by M2-like Mφ are more frequently present in stable plaques as major anti-inflammatory mediators (54). Studies have shown that blocking core fucosylation, downstream of TGF-β, can reduce markers of renal fibrosis. Although the mechanisms of renal fibrosis and AVF dysfunction differ, there is significant overlap. In addition, changes in glycosylation have been shown to affect cell proliferation, transformation, migration, and apoptosis (28). Arg-1 promotes VSMC proliferation and inhibits inflammation: M2-like Mφ infiltration plays a key role in regulating VSMCS proliferation and vascular wall thickening during AVF maturation, so Arg-1 may be involved in AVF maturation; However, in the rat model of common carotid artery injury, Arg-1 also promotes the formation of NIH, and therefore, Arg-1 may have an adverse effect on AVF function maintenance. However, arterial remodeling is different from AVF, Arg-1 may play a critical regulatory role in the balance between vascular wall thickening and NIH formation, and thus may be beneficial or harmful in AVF function maintenance (55).

When inflammation occurs, Mφ polarizes into M1-like Mφ, producing pro-inflammatory mediators that effectively control and clear infection and remove dead cells, but can lead to tissue damage if the M1 phase persists. Therefore, M2-like Mφ secretes a large amount of anti-inflammatory mediators, inhibits inflammatory responses, promotes tissue repair, vascular remodeling, and maintains homeostasis (37). For example, in a mouse model of CKD with AVF, iNOS, a marker of pro-inflammatory M1-like Mφ, was first increased, followed by Arg-1, a marker of anti-inflammatory M2-like Mφ (56). Thus, the balance of M1-like Mφ and M2-like Mφ polarization determines the fate of the organ in inflammation or injury. Revealing the polarization and recruitment process of M1-like Mφ and M2-like Mφ may provide a new therapeutic approach for regulating the balance of phenotype, quantity and distribution of vascular remodeling in AVF.

### T lymphocyte: function by regulating Mφ

T lymphocyte plays a role in acquired immunity and are also involved in the regulation of inflammation. APC processing antigens are presented to major histocompatibility complex (MHC) molecules, which then activate CD8 ^+^ T lymphocyte with MHC- I molecules, and CD4^+^ T lymphocyte with MHC - II molecules (48, 57). After activation, CD8^+^T lymphocyte has antigen-specific cytotoxicity, also known as cytotoxic T lymphocyte (CTL) (58). Unlike CD8^+^T lymphocyte, CD4^+^T lymphocyte activates and differentiates into different subsets, distinguished by the production of specific cytokine and effector functions, including helper (Th) and regulatory T (Treg) lymphocytes (59). CD4^+^ lymphocyte and CD8^+^T lymphocyte play a major role in the initiation and continuation of the inflammatory cascade, and can also regulate other immune cells through the inflammatory response, such as secreting cytokines to regulate the inflammatory state of Mφ (34). In AVF of athymic rnu nude rats lacking mature T lymphocytes and euthymic control animals, the presence of CD4^+^T lymphocyte was consistent with the presence of Mφ, and the absence of mature T lymphocyte showed a tendency of decreasing Mφ infiltration (60). Therefore, T lymphocyte regulate the accumulation of Mφ in mature venous walls, thereby controlling adaptive remodeling (50).

#### Helper (Th)T lymphocyte

Th lymphocyte can be divided into different subgroups according to the type of cytokine they secrete: Th1 lymphocyte has a pro-inflammatory effects, and Th2 lymphocyte has an anti-inflammatory effects. Analysis of clinical specimens of atherosclerosis and graft atherosclerosis vessels shows that in the pathogenesis of T lymphocyte mediated vascular injury and remodeling, the Th1-dominant response leads to intimal thickening and lumen stenosis, while the Th2-dominant response leads to arterial dilation and lumen ectasia (61).

Th1 lymphocyte produces pro-inflammatory cytokines such as IFN-γ, IL-2 and TNF-β. However, the direction of Th lymphocyte differentiation is influenced by different cytokines and chemokines, especially IL-12. IFN-γ is the signature cytokine of Th1 lymphocyte and can be promoted by IL-12. Promote Th1 lymphocyte differentiation by increasing IL-12 production and inhibit Th2 lymphocyte differentiation by inhibiting IL-4 (62). Studies have shown that the percentage and absolute number of activated CD4^+^T lymphocyte in peripheral blood produce IFN-γ are higher in patients with hypertension than in patients with normal blood pressure (32). There is a positive feedback relationship between IFN-γ and IL-12 production and Th1 lymphocyte differentiation, making Th lymphocyte differentiation balance closer to Th1 lymphocyte differentiation. The cumulative effects of these cytokines promote the transformation of the immune microenvironment from anti-inflammatory to pro-inflammatory, resulting in the accumulation of inflammatory cells and their secretions and inhibiting AVF maturation. Th2 lymphocyte produces anti-inflammatory cytokines such as IL-4, IL-5, IL-10 and IL-13. IL-4 is the signature cytokine of Th2 lymphocyte, which can promote Th2 lymphocyte differentiation and up-regulate the expression of IL-5, thus inhibiting Th1 lymphocyte differentiation and IFN-γ production. This cascade promotes the resolution of inflammation, which promotes the maturation of AVF (16). In addition, Th lymphocyte can regulate the inflammatory state of Mφ. Th1 lymphocyte promotes the polarization of Mφ toward the proinflammatory phenotype and activate other inflammatory cells. Th2 lymphocyte promotes Mφ polarization to the anti-inflammatory phenotype (63, 64). In atherosclerosis, IFN-γ destabilizes plaque stability and recruits Mφ to promote polarization of the M1-like phenotype (65). Studies have shown that M2-like Mφ is required for AVF maturation. taken together, Th2 lymphocyte may promote AVF maturation by inhibiting Th1 lymphocyte differentiation and inducing M2-like Mφ polarization (53).

#### Regulatory T (Treg) lymphocyte

Treg lymphocyte is anti-inflammatory T lymphocyte characterized by CD25 and Foxp3 labeling, secreting anti-inflammatory cytokines such as IL-10, IL-35 and TGF -β, inhibiting innate and acquired immune responses, regulating the duration and amplitude of inflammatory responses, and thus maintaining immune tolerance and immune homeostasia (66). Treg lymphocyte secretes anti-inflammatory cytokines to inhibite inflammatory cells including not only Th1 lymphocyte, CTL and M1-like Mφ, but also inhibit anti-inflammatory Th2 lymphocyte and induce M2-like Mφ polarization of anti-inflammatory cells, promote inflammation lysis, tissue healing and vascular remodeling, thus promote AVF maturation (67). In CD4^+^T lymphocyte, IL-10 is thought to be produced primarily by Treg lymphocyte. Uncontrolled secretion of IFN-γ by Th1 lymphocyte may lead to severe tissue damage, and inhibition of the spatio-temporal dependent balance between IFN-γ secretion by Th1 lymphocyte and IL-10 secretion by Treg lymphocyte may enable an effective immune response with limited tissue damage (68, 69). However, due to reduced IFN-γ production, recruitment of monocytes and effector T lymphocyte in the lesion was also reduced (70). These studies suggest that depletion of Th1 lymphocyte is induced by Treg lymphocyte and promotes AVF maturation (71). TGF-β inhibited the production of MCP-1, and the secretion of proinflammatory cytokines was significantly reduced after monocytes were treated with Treg lymphocyte (16). Treg lymphocyte expressed surface marker such as co-stimulator, CD4 ^+^ CD25 ^+^ FoxP3 ^+^, and these elevated markers maintened the mechanism of inhibiting inflammation, and promoted AVF maturation (72). IL-2 helps maintain Foxp3 expression through IL-2Rα/CD25 signaling, thereby promoting Treg lymphocyte function (73). Co-stimulator takes part in IL-10-mediated inhibition of effector T lymphocyte, lack of Co-stimulator can lead to decrease in the number and function of Foxp3 Treg lymphocyte, and accelerate the progression of atherosclerosis (65). The accumulation of inflammatory immune cells on the blood vessel wall leads to thickening of intima and inflammation, which leads to lumen narrowing and thrombosis (74). By regulating Treg lymphocyte, the balance between effector T lymphocyte and Treg lymphocyte is coordinated to promote anti-inflammatory changes in the blood vessel wall, thereby inhibiting the immune response and promoting AVF maturation (67).

#### Cytotoxic T Lymphocyts (CTL)

CTL lymphocytes is a type of T lymphocyte characterized by CD8^+^ labeling and secrete perforin, granzyme B(GzmB), IFN-g, and TNF-a. Perforin forms pores in the target cell membrane, through which granzyme can enter and induce apoptosis (75). CTL lymphocyte is more cytotoxic than CD4^+^T lymphocyte, can induce vascular wall apoptosis, and inhibit immune cell function under certain conditions (60, 76). Unlike CD4^+^ T lymphocyte, the function of CD8^+^ T lymphocyte does not depend on Mφ. Studies have shown that CD8^+^ T lymphocyte is locally activated after vein graft surgery in CD4^+^ or CD8^+^ T lymphocytopenia mice, and play an important role in protecting the patency of vein graft. When CD8^+^ T lymphocyte was present, the graft vein was unobstructed. However, CD8^+^ T lymphocyte was lacking, graft occlusion was observed, and apoptosis was increased. Therefore, CD8^+^T lymphocyte can reduce NIH and improve graft vein patency (77). In atherosclerosis, CD8^+^T lymphocyte mediated VSMC death leads to plaque instability, but CD8^+^T lymphocyte induced apoptosis limits NIH after arterial injury (74). However, most of the CD8^+^T lymphocyte on the venous wall were inactivated, suggesting that CD8^+^T lymphocyte may not be the primary regulatory cells of venous remodeling (51).

### Dendritic cell (DC)

DC generally belongs to a class of innate lymphoid cell, defined as cell that express high levels of MHC II and the cell surface integrin CD11c (78, 79). DC is a professional antigen-presenting cell (APC) with antigen-presenting, pro-inflammatory, and anti-inflammatory functions to initiate antigen-specific immune responses, induce immune tolerance, and regulate immune homeostasis. It plays a role in both innate and adaptive immunity (16). DC acts as an APC to control the activation and phenotype of T lymphocyte (80). In the AVF formation region, chronically activated DC continues to express CD83 and CD86, and these surface molecules act as co-stimulatory molecules to activate other DC, thereby activating naive T lymphocyte to become effector T lymphocyte, promoting effector T lymphocyte proliferation and harmful T lymphocytes apoptosis (16). The appearance of DC in atherosclerosis is similar, and the stability of plaque is negatively correlated with the accumulation of DC, suggesting that DC may regulate local inflammatory response and promote plaque vulnerability (16, 81). Blocking CD11c^+^ activity during plaque formation may have therapeutic effects (80). Fasin^+^ is a surface marker of mature DC, while human leukocyte antigen (HLA-DR) and trigger receptor expressed on myeloid cells-1 (TREM-1) are surface markers of associated autoimmune diseases and indicators of progressive inflammation, respectively (69). In AVF with chronic, unresolved inflammation, these markers are found to be upregulated (16). Reducing the expression of HLA-DR, and TREM-1, CD83 and CD86, is a multifactorial anti-inflammatory therapeutic mechanism of DC during AVF maturation (16). Central chemokine receptor 7 (CCR7) is a chemokine of CD11c^+^. Studies have shown that high expression of CCR7 can lead to increased DC migration, subsequent thrombolysis, and promote AVF maturation (16). DC controls excessive inflammation in acquired immunity by secreting anti-inflammatory cytokines IL-10 and TGF-β (82). TGF-β inhibited the proliferation, activation and differentiation of pro-inflammatory T lymphocyte, and promoted the apoptosis of pro-inflammatory T lymphocyte. However, studies have shown that interfering with TGF-β type II receptor signaling in CD11c^+^ promotes the formation of atherosclerotic lesions and the expansion of activating effect T lymphocyte (80). These results suggest that DC-T lymphocyte interaction and anti-inflammatory function of DC play a role in AVF maturation and functional maintenance, and that DC can target different stages of AVF vascular remodeling, and can be used to help regulate AVF vascular remodeling in the clinic.

### Neutrophil

Neutrophil (NEUT) is myeloid white blood cells that accounting for 50%-70% of all circulating white blood cells (83). It is a component of the innate immune system and can also regulate the immune microenvironment through direct intercellular contact or soluble media communication with Mφ, DC, T lymphocyte, and B lymphocyte (83, 84). Traditionally, NEUT was thought to be present only in the acute phase of inflammation and only to play a role in eliminating pathogens. However, studies have shown that the function of NEUT is not limited to infection, it also plays a role in different types of inflammation (infection and sterility) (85, 86). NEUT synthesises and releases a variety of grain proteins, including MMP, neutrophil elastase (NE), lactoferrin, and pro-inflammatory and anti-inflammatory cytokines (86). Researches have shown that when EC and SMC are co-cultured and exposed to increased shear stress in AVF, gene expression of lactoferrin increases (87). Misra et al (88) investigated the change of protein expression in venous stenosis was studied by protein spectrometry, and lactoferrin expression was significantly increased. In a study of late AVF failure in chronic HD patients, NE and lactoferrin levels were positively correlated with AVF stenosis, and elevated NE levels were also found to be an independent predictor of late AVF failure (18). The neutrophil-lymphocyte ratio (NLR), defined as the absolute number of NEUT divided by the absolute number of lymphocytes, is a strong inflammatory indicator similar to MLR and is associated with coronary atherosclerosis and restenosis (89, 90). In another study of late AVF failure in chronic HD patients, AVF stenosis was found that patients with elevated NLR levels, and the severity of stenosis was positively correlated with NLR levels. It was also found that elevated NLR levels were an independent predictor of late AVF failure (91). In studies of AVF maturation, NLR measured in the blood before surgery was found to be a potential biomarker for predicting AVF maturation. This NLR-associated AVF maturation may be due to the necessary early local pro-inflammatory response in the vascular wall (92). In addition to being a pro-inflammatory cell in cardiovascular disease, there is evidence that NEUT also contributes to tissue repair (93). In an experimental model of restenosis, blocking NEUT in diseased arteries promoted the development of NIH and inhibited damaged intima repair (94). NEUT and its granuloprotein are believed to guide immune cells, especially monocytes, into atherosclerotic lesions and directly affect the occurrence of atherogenesis (95). In a rabbit model of arterial balloon injury, the infiltration of NEUT prior to NIH was found, but no Mφ was found (38). In a mouse model of vascular inflammation, NEUT-secreted chemoattractant proteins can immobilize EC and induce firm monocyte adhesion (96). In addition, granuloprotein directly activates Mφ in human and mice, inducing the secretion of pro-inflammatory cytokines that have been shown to promote atherosclerosis (93). Studies have shown that in patients with various cardiovascular diseases, NE levels in the blood are elevated, NE is abundant in human atherosclerotic plaques, and that NE/proteinase 3 double knockout mice reduced atherosclerotic lesions (97–99). These findings suggest that NEUT plays a role in vascular remodeling in AVF.

### B lymphocyte

**Figure 1:**
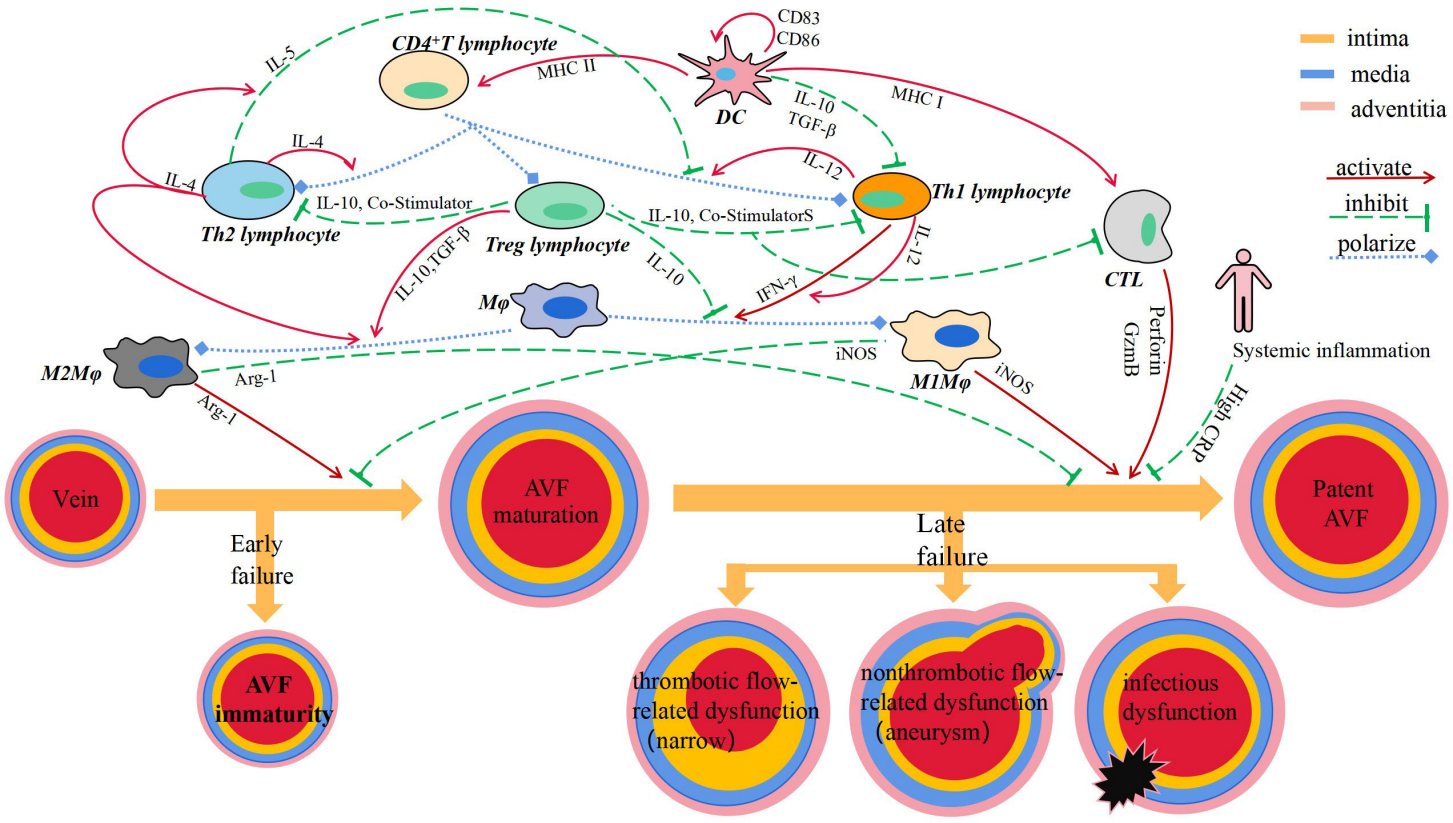
Immune cells and secreted cytokines are involved in vascular remodeling of AVF.

By reviewing the relevant literature, it has not been found that B lymphocyte plays a role in vascular remodeling of AVF (**Figure 1**).

## Drug treatment strategies targeting immune cells to regulate inflammation

Immunotherapy can promote the maturation and functional maintenance of AVF. Immunotherapy refers to the treatment of disease by activating or suppressing the immune system, which is characterized by altering the infiltration of immune cells and the expression of related cytokines (100). Most immunomodulators belong to biological drugs, which are characterized by their large volume, low stability, poor permeability to the lesion site, and limited ability to cross cell membranes. However, AVF anastomosis is a local vascular injury that must be treated effectively with a high enough concentration of the drug at the anastomosis (36). Local delivery systems can be used for high anastomotic concentrations with minimal systemic toxicity. Administration strategies include direct administration to the endothelium, administration to the adventitia, administration to the entire vessel wall, or the use of mechanical support devices (22). The drug delivery methods of liposomes include hydrogels, living cells, particles, inorganic materials, polymeric micelles, drug crystal and carrier proteins (101).

### Targeting macrophage regulates inflammation

In addition to their LDL cholesterol lowering effects, statins have anti-inflammatory effects (102), and are composed of cyclodextrin particles (MP) with reliable and sustained drug release properties. Clinical studies have shown that the use of high-dose statins reduces the risk of AVF failure and can improve AVF patency (103). Studies have shown that in the mouse (C57Bl/6J) AVF model, simvastatin (SV)-loaded cyclodextrin polymer (CDP) intervention can inhibit the accumulation of Mφ in the intima tissue of AVF outflow tract, reduce the gene expression of vascular endothelial growth factor (VEGF)-A and MMP-9, improve vascular remodeling, and reduce the formation of NIH. Thus, it can promote the maturation of AVF and maintain its patency (104, 105).

Prednisolone is a commonly used anti-inflammatory drug in clinic. Liposomes have been shown to facilitate the selective delivery of drugs to inflammatory tissues with highly permeable microvessels that are then engulfed by Mφ in the inflammatory tissue (106). Therefore, liposomes drugs can selectively target AVF after anastomosis, which is often the cause of AVF failure. The mouse AVF model showed that prednisolone was coated with lipids to form liposomal prednisolone (L-Pred), which was swallowed by Mφ after intravenous injection, reduced the infiltration of Mφ, inhibited the inflammatory response and MMP activity in Mφ, alleviated the local inflammatory response of mouse AVF, stimulated outward remodeling, and promoted the maturation of AVF (40).

Human resistance CX3CR1 VHH molecule (CX3CR1 VHH, BI, 655088), CX3CR1 is a specific receptor fractalkine, belonging to the chemokine receptor superfamily. CX3CR1, expressed in monocytes, mediates the infiltration of Mφ into blood vessels, and is increased in clinical AVF specimens with stenosis and in mouse models of CKD with AVF. In a humanized mouse AVF model carried human CX3CR1 gene, inhibition of CX3CR1 with anti-human CX3CR1 VHH molecule reduced Mφ aggregation and procytokine production, promoted vascular remodeling and decreased NIH (107), and promoted AVF maturation and function maintenance.

Sirolimus can inhibit the proliferation of Mφ and the phosphorylation of Akt1-mTORC1 signaling pathway in Mφ at the mature stage of AVF, resulting in the continuous reduction of VSMC in late AVF (39). Sirolimus can also induce the expression of Foxp3, promote the differentiation and upregulation of Treg lymphocytes, which secrete IL-10 and TGF-β, inhibit the inflammation associated with AVF failure, and help improve the AVF patency (108). Thus, sirolimus promotes AVF maturation and functional maintenance while reducing vessel wall thickening, but has no effect on vessel diameter expansion. Since sirolimus in clinically used in coronary stents to protect lumen diameter and reduce restenosis after stenting. Sirolimus should reduce the NIH potential in AVF (109).

### Targeting T lymphocyte regulates inflammation

The combination of tacrolimus and sirolimus may be more effective in reducing the incidence of vascular stenosis, which may be related to the fact that tacrolimus specifically acts on T lymphocyte while sirolimus inhibits Mφ.

Cyclosporine A (CsA) can inhibit the function of T lymphocyte and reduce the accumulation of Mφ, thereby regulating venous adaptive remodeling and promoting AVF maturation. In mouse models of AVF, CsA selectively blocked the proliferation and differentiation of CD4^+^T lymphocyte, thereby inhibiting INF-γ and IL-2 secretion, which are necessary for other inflammatory cells, including Mφ, and ultimately leading to reduced vessel wall thickening and increased AVF outward remodeling in wild-type mice. However, these effects were eliminated in T lymphocyte deficient nude mice, indicating that the effect of CsA on Mφ aggregation and adaptive remodeling is T lymphocyte (50).

Programmed death ligand (PD-L)-1, a ligand that binds to programmed cell death (PD)-1, is specifically expressed on T lymphocyte, thereby increasing Treg lymphocyte and M2-like Mφ and decreasing Th lymphocyte and M1-like Mφ. In a mouse model of aortic - inferior vena cava AVF, administration of anti-PD-L1 antibodies inhibited PD-L1 activity, thickened AVF wall, increased thrombosis, and reduced AVF patency. The effect of anti-PD-L1 antibody on T lymphocyte deficient nude mice was weakened, but T lymphocyte transplantation could restore it, suggesting that the effect of anti-PD-L1 antibody on venous remodeling is also dependent on T lymphocyte. The above studies suggest that PD-L1 can specifically regulate T lymphocyte, and T lymphocyte can regulate Mφ in venous remodeling, thereby increasing the thickness of blood vessel wall and the patency of AVF, thereby promoting AVF vascular remodeling (51) (**Table 1**).

**Table 1:** Drug treatment strategies targeting immune cells to regulate inflammation.

Drug	Clinical trials and results
Targeting macrophage regulates inflammation	
Statin	The use of high-dose statins reduces the risk of AVF failure and can improve AVF patency
Prednisolone	–
anti-human CX3CR1 VHH molecule	–
Sirolimus	Sirolimus in clinically used in coronary stents to protect lumen diameter and reduce restenosis after stenting.
Targeting T lymphocyte regulates inflammation	
Tacrolimus	The combination of tacrolimus and sirolimus may be more effective in reducing the incidence of vascular stenosis
Cyclosporine A	–
Programmed death ligand-1	–

## Conclusion

In summary, CD4^+^T lymphocyte and Mφ are important regulators during AVF maturation and function maintenance. CD8^+^T lymphocyte, NUET and DC are also involved. However, due to the diversity of immune cell functions, many of their roles in AVF maturation and functional maintenance are unknown. Current evidence suggests that Th2 lymphocyte contributes to increase blood flow and outward remodeling during AVF maturation, and M2-like Mφ contributes to venous wall thickening; In AVF function maintenance, Th1 lymphocyte and M1φ involved in decreasing NIH and promoting long-term patency of AVF. Th2 lymphocyte, Treg lymphocyte, and M2-like Mφ are required for AVF maturation and are associated with AVF, narrowing; Both Th1 lymphocyte and M1-like Mφ are associated with immaturity of AVF and also promote the maintenance of AVF function. Therefore, maintaining the function of targeted immune cells, especially CD4^+^ T lymphocyte and Mφ, is of great significance for AVF reconstitution during AVF maturation.

## Author contributions

YZ: Writing – original draft. XK: Writing – original draft. LL: Writing – review & editing. DX: Writing – review & editing, Conceptualization.

## References

[1] Wang L Xu X Zhang M Hu C Zhang X Li C. Prevalence of chronic kidney disease in China: results from the sixth China chronic disease and risk factor surveillance. JAMA Intern Med. (2023) 183:298–310. doi: 10.1001/jamainternmed.2022.6817 36804760 PMC9941971

[2] Murphy D McCulloch CE Lin F Banerjee T Bragg-Gresham JL Eberhardt MS. Trends in prevalence of chronic kidney disease in the United States. Ann Intern Med. (2016) 165:473–81. doi: 10.7326/M16-0273 PMC5552458 27479614

[3] Santoro D Benedetto F Mondello P Pipitò N Barillà D Spinelli F. Vascular access for hemodialysis: current perspectives. Int J Nephrol Renovasc Dis. (2014) 7:281–94. doi: 10.2147/IJNRD.S46643 PMC4099194 25045278

[4] Huber TS Berceli SA Scali ST Neal D Anderson EM Allon M. Arteriovenous fistula maturation, functional patency, and intervention rates. JAMA Surg. (2021) 156:1111–8. doi: 10.1001/jamasurg.2021.4527 PMC8459303 34550312

[5] Al-Jaishi AA Oliver MJ Thomas SM Lok CE Zhang JC Garg AX. Patency rates of the arteriovenous fistula for hemodialysis: a systematic review and meta-analysis. Am J Kidney Dis. (2014) 63:464–78. doi: 10.1053/j.ajkd.2013.08.023 24183112

[6] Kong X Tang L Liang L Cao W Zhang L Yong W. Clinical outcomes following the surgery of new autologous arteriovenous fistulas proximal to the failed ones in end-stage renal disease patients: a retrospective cohort study. Ren Fail. (2019) 41:1036–44. doi: 10.1080/0886022X.2019.1696210 PMC6913653 31814501

[7] Rothuizen TC Wong C Quax PH van Zonneveld AJ Rabelink TJ Rotmans JI. Arteriovenous access failure: more than just intimal hyperplasia? Nephrol Dial Transplant. (2013) 28:1085–92. doi: 10.1093/ndt/gft068 23543595

[8] Kaygin MA Halici U Aydin A Dag O Binici DN Limandal HK. The relationship between arteriovenous fistula success and inflammation. Ren Fail. (2013) 35:1085–8. doi: 10.3109/0886022X.2013.815100 23906289

[9] Cunnane CV Cunnane EM Walsh MT. A review of the hemodynamic factors believed to contribute to vascular access dysfunction. Cardiovasc Eng Technol. (2017) 8:280–94. doi: 10.1007/s13239-017-0307-0 28527110

[10] Chiu JJ Chien S. Effects of disturbed flow on vascular endothelium: pathophysiological basis and clinical perspectives. Physiol Rev. (2011) 91:327–87. doi: 10.1152/physrev.00047.2009 PMC3844671 21248169

[11] Xiao Y Vazquez-Padron RI Martinez L Singer HA Woltmann D Salman LH. Role of platelet factor 4 in arteriovenous fistula maturation failure: What do we know so far? J Vasc Access. (2024) 25:390–406. doi: 10.1177/11297298221085458 35751379 PMC9974241

[12] Henderson NC Rieder F Wynn TA. Fibrosis: from mechanisms to medicines. Nature. (2020) 587:555–66. doi: 10.1038/s41586-020-2938-9 PMC8034822 33239795

[13] Martinez L Duque JC Tabbara M Paez A Selman G Hernandez DR. Fibrotic venous remodeling and nonmaturation of arteriovenous fistulas. J Am Soc Nephrol. (2018) 29:1030–40. doi: 10.1681/ASN.2017050559 PMC5827597 29295872

[14] Applewhite B Gupta A Wei Y Yang X Martinez L Rojas MG. Periadventitial beta-aminopropionitrile-loaded nanofibers reduce fibrosis and improve arteriovenous fistula remodeling in rats. Front Cardiovasc Med. (2023) 10:1124106. doi: 10.3389/fcvm.2023.1124106 36926045 PMC10011136

[15] Du J Liang L Liu S Yang X Cao S Zhang H. Neointimal hyperplasia in the inferior vena cava of adenine-induced chronic kidney disease rats with aortocaval fistulas. Clin Exp Nephrol. (2020) 24:1007–14. doi: 10.1007/s10157-020-01927-3 32666345

[16] Samra G Rai V Agrawal DK. Innate and adaptive immune cells associate with arteriovenous fistula maturation and failure. Can J Physiol Pharmacol. (2022) 100:716–27. doi: 10.1139/cjpp-2021-0731 35671528

[17] Matsubara Y Kiwan G Fereydooni A Langford J Dardik A. Distinct subsets of T cells and macrophages impact venous remodeling during arteriovenous fistula maturation. JVS Vasc Sci. (2020) 1:207–18. doi: 10.1016/j.jvssci.2020.07.005 PMC7971420 33748787

[18] Oh DJ Lee JH Kwon YE Choi HM. Relationship between arteriovenous fistula stenosis and circulating levels of neutrophil granule proteins in chronic hemodialysis patients. Ann Vasc Surg. (2021) 77:226–35. doi: 10.1016/j.avsg.2021.05.056 34437969

[19] Gameiro J Ibeas J. Factors affecting arteriovenous fistula dysfunction: A narrative review. J Vasc Access. (2020) 21:134–47. doi: 10.1177/1129729819845562 31113281

[20] Lee T Mokrzycki M Moist L Maya I Vazquez M Lok CE. Standardized definitions for hemodialysis vascular access. Semin Dial. (2011) 24:515–24. doi: 10.1111/j.1525-139X.2011.00969.x PMC3999346 21906166

[21] Lok CE Huber TS Lee T Shenoy S Yevzlin AS Abreo K. KDOQI clinical practice guideline for vascular access: 2019 update. Am J Kidney Dis. (2020) 75:S1–S164. doi: 10.1053/j.ajkd.2019.12.001 32778223

[22] Hu H Patel S Hanisch JJ Santana JM Hashimoto T Bai H. Future research directions to improve fistula maturation and reduce access failure. Semin Vasc Surg. (2016) 29:153–71. doi: 10.1053/j.semvascsurg.2016.08.005 PMC5547899 28779782

[23] Tronc F Mallat Z Lehoux S Wassef M Esposito B Tedgui A. Role of matrix metalloproteinases in blood flow-induced arterial enlargement: interaction with NO. Arterioscler Thromb Vasc Biol. (2000) 20:E120–126. doi: 10.1161/01.atv.20.12.e120 11116076

[24] Chan CY Chen YS Ma MC Chen CF. Remodeling of experimental arteriovenous fistula with increased matrix metalloproteinase expression in rats. J Vasc Surg. (2007) 45:804–11. doi: 10.1016/j.jvs.2006.12.063 17398390

[25] Hu K Guo Y Li Y Lu C Cai C Zhou S. Oxidative stress: An essential factor in the process of arteriovenous fistula failure. Front Cardiovasc Med. (2022) 9:984472. doi: 10.3389/fcvm.2022.984472 36035909 PMC9403606

[26] Tellides G Pober JS. Inflammatory and immune responses in the arterial media. Circ Res. (2015) 116:312–22. doi: 10.1161/CIRCRESAHA.116.301312 25593276

[27] Roy-Chaudhury P Kruska L. Future directions for vascular access for hemodialysis. Semin Dial. (2015) 28:107–13. doi: 10.1111/sdi.12329 25482103

[28] Brahmbhatt A Remuzzi A Franzoni M Misra S. The molecular mechanisms of hemodialysis vascular access failure. Kidney Int. (2016) 89:303–16. doi: 10.1016/j.kint.2015.12.019 PMC4734360 26806833

[29] Hofstra L Tordoir JH Kitslaar PJ Hoeks AP Daemen MJ. Enhanced cellular proliferation in intact stenotic lesions derived from human arteriovenous fistulas and peripheral bypass grafts. Does it correlate with flow parameters? Circulation. (1996) 94:1283–90. doi: 10.1161/01.cir.94.6.1283 8822981

[30] Ross R. The pathogenesis of atherosclerosis: a perspective for the 1990s. Nature. (1993) 362:801–9. doi: 10.1038/362801a0 8479518

[31] Rai V Agrawal DK. The role of damage- and pathogen-associated molecular patterns in inflammation-mediated vulnerability of atherosclerotic plaques. Can J Physiol Pharmacol. (2017) 95:1245–53. doi: 10.1139/cjpp-2016-0664 28746820

[32] Petrie JR Guzik TJ Touyz RM. Diabetes, hypertension, and cardiovascular disease: clinical insights and vascular mechanisms. Can J Cardiol. (2018) 34:575–84. doi: 10.1016/j.cjca.2017.12.005 PMC5953551 29459239

[33] Bezhaeva T Wong C de Vries MR van der Veer EP van Alem CMA Que I. Deficiency of TLR4 homologue RP105 aggravates outward remodeling in a murine model of arteriovenous fistula failure. Sci Rep. (2017) 7:10269. doi: 10.1038/s41598-017-10108-4 28860634 PMC5578984

[34] Mukhopadhyay S Gabre J Chabasse C Bromberg JS Antalis TM Sarkar R. Depletion of CD4 and CD8 positive T cells impairs venous thrombus resolution in mice. Int J Mol Sci. (2020) 21:1650. doi: 10.3390/ijms21051650 32121269 PMC7084232

[35] Idris-Khodja N Mian MO Paradis P Schiffrin EL. Dual opposing roles of adaptive immunity in hypertension. Eur Heart J. (2014) 35:1238–44. doi: 10.1093/eurheartj/ehu119 PMC4019914 24685711

[36] Lee T Misra S. New insights into dialysis vascular access: molecular targets in arteriovenous fistula and arteriovenous graft failure and their potential to improve vascular access outcomes. Clin J Am Soc Nephrol. (2016) 11:1504–12. doi: 10.2215/CJN.02030216 PMC4974876 27401527

[37] Shapouri-Moghaddam A Mohammadian S Vazini H Taghadosi M Esmaeili SA Mardani F. Macrophage plasticity, polarization, and function in health and disease. J Cell Physiol. (2018) 233:6425–40. doi: 10.1002/jcp.26429 PMC13482560 29319160

[38] Welt FG Edelman ER Simon DI Rogers C. Neutrophil, not macrophage, infiltration precedes neointimal thickening in balloon-injured arteries. Arterioscler Thromb Vasc Biol. (2000) 20:2553–8. doi: 10.1161/01.atv.20.12.2553 11116052

[39] Guo X Fereydooni A Isaji T Gorecka J Liu S Hu H. Inhibition of the akt1-mTORC1 axis alters venous remodeling to improve arteriovenous fistula patency. Sci Rep. (2019) 9:11046. doi: 10.1038/s41598-019-47542-5 31363142 PMC6667481

[40] Wong C Bezhaeva T Rothuizen TC Metselaar JM de Vries MR Verbeek FP. Liposomal prednisolone inhibits vascular inflammation and enhances venous outward remodeling in a murine arteriovenous fistula model. Sci Rep. (2016) 6:30439. doi: 10.1038/srep30439 27460883 PMC4962038

[41] Gorecka J Fereydooni A Gonzalez L Lee SR Liu S Ono S. Molecular targets for improving arteriovenous fistula maturation and patency. Vasc Investig Ther. (2019) 2:33–41. doi: 10.4103/VIT.VIT_9_19 PMC6788624 31608322

[42] Du Cheyne C Tay H De Spiegelaere W. The complex TIE between macrophages and angiogenesis. Anat Histol Embryol. (2020) 49:585–96. doi: 10.1111/ahe.12518 31774212

[43] Hu S Wang D Ma T Yuan F Zhang Y Gao X. Association between preoperative monocyte-to-lymphocyte ratio and late arteriovenous fistula dysfunction in hemodialysis patients: A cohort study. Am J Nephrol. (2021) 52:854–60. doi: 10.1159/000519822 34749361

[44] Brahmbhatt A NievesTorres E Yang B Edwards WD Roy Chaudhury P Lee MK. The role of Iex-1 in the pathogenesis of venous neointimal hyperplasia associated with hemodialysis arteriovenous fistula. PloS One. (2014) 9:e102542. doi: 10.1371/journal.pone.0102542 25036043 PMC4103828

[45] Shih CM Huang CY Liao LR Hsu CP Tsao NW Wang HS. Nickel ions from a corroded cardiovascular stent induce monocytic cell apoptosis: Proposed impact on vascular remodeling and mechanism. J Formos Med Assoc. (2015) 114:1088–96. doi: 10.1016/j.jfma.2014.03.007 24840272

[46] Roy-Chaudhury P Khan R Campos B Wang Y Kurian M Lee T. Pathogenetic role for early focal macrophage infiltration in a pig model of arteriovenous fistula (AVF) stenosis. J Vasc Access. (2014) 15:25–8. doi: 10.5301/jva.5000151 24043320

[47] Wang X Khalil RA. Matrix metalloproteinases, vascular remodeling, and vascular disease. Adv Pharmacol. (2018) 81:241–330. doi: 10.1016/bs.apha.2017.08.002 29310800 PMC5765875

[48] McMaster WG Kirabo A Madhur MS Harrison DG. Inflammation, immunity, and hypertensive end-organ damage. Circ Res. (2015) 116:1022–33. doi: 10.1161/CIRCRESAHA.116.303697 PMC4535695 25767287

[49] Sica A Mantovani A. Macrophage plasticity and polarization: *in vivo* veritas. J Clin Invest. (2012) 122:787–95. doi: 10.1172/JCI59643 PMC3287223 22378047

[50] Matsubara Y Kiwan G Liu J Gonzalez L Langford J Gao M. Inhibition of T-cells by cyclosporine A reduces macrophage accumulation to regulate venous adaptive remodeling and increase arteriovenous fistula maturation. Arterioscler Thromb Vasc Biol. (2021) 41:e160–74. doi: 10.1161/ATVBAHA.120.315875 PMC7904667 33472405

[51] Matsubara Y Gonzalez L Kiwan G Liu J Langford J Gao M. PD-L1 (Programmed death ligand 1) regulates T-cell differentiation to control adaptive venous remodeling. Arterioscler Thromb Vasc Biol. (2021) 41:2909–22. doi: 10.1161/ATVBAHA.121.316380 PMC8664128 34670406

[52] Tang D Chen S Hou D Gao J Jiang L Shi J. Regulation of macrophage polarization and promotion of endothelialization by NO generating and PEG-YIGSR modified vascular graft. Mater Sci Eng C Mater Biol Appl. (2018) 84:1–11. doi: 10.1016/j.msec.2017.11.005 29519417

[53] Kuwahara G Hashimoto T Tsuneki M Yamamoto K Assi R Foster TR. CD44 promotes inflammation and extracellular matrix production during arteriovenous fistula maturation. Arterioscler Thromb Vasc Biol. (2017) 37:1147–56. doi: 10.1161/ATVBAHA.117.309385 PMC5467640 28450292

[54] Yang S Yuan HQ Hao YM Ren Z Qu SL Liu LS. Macrophage polarization in atherosclerosis. Clin Chim Acta. (2020) 501:142–6. doi: 10.1016/j.cca.2019.10.034 31730809

[55] Peyton KJ Ensenat D Azam MA Keswani AN Kannan S Liu XM. Arginase promotes neointima formation in rat injured carotid arteries. Arterioscler Thromb Vasc Biol. (2009) 29:488–94. doi: 10.1161/ATVBAHA.108.183392 PMC2662760 19164802

[56] Miki K Kumar A Yang R Killeen ME Delude RL. Extracellular activation of arginase-1 decreases enterocyte inducible nitric oxide synthase activity during systemic inflammation. Am J Physiol Gastrointest Liver Physiol. (2009) 297:G840–848. doi: 10.1152/ajpgi.90716.2008 PMC2763806 19713467

[57] Konjar S Veldhoen M. Dynamic metabolic state of tissue resident CD8 T cells. Front Immunol. (2019) 10:1683. doi: 10.3389/fimmu.2019.01683 31379871 PMC6650586

[58] Larosa DF Orange JS. 1. Lymphocytes. J Allergy Clin Immunol. (2008) 121:S364–369. doi: 10.1016/j.jaci.2007.06.016 18241683

[59] Weaver CT Harrington LE Mangan PR Gavrieli M Murphy KM. Th17: an effector CD4 T cell lineage with regulatory T cell ties. Immunity. (2006) 24:677–88. doi: 10.1016/j.immuni.2006.06.002 16782025

[60] Duque JC Martinez L Mesa A Wei Y Tabbara M Salman LH. CD4(+) lymphocytes improve venous blood flow in experimental arteriovenous fistulae. Surgery. (2015) 158:529–36. doi: 10.1016/j.surg.2015.02.018 PMC4492885 25999254

[61] Wang Y Burns WR Tang PC Yi T Schechner JS Zerwes HG. Interferon-gamma plays a nonredundant role in mediating T cell-dependent outward vascular remodeling of allogeneic human coronary arteries. FASEB J. (2004) 18:606–8. doi: 10.1096/fj.03-0840fje 14734640

[62] Ou HX Guo BB Liu Q Li YK Yang Z Feng WJ. Regulatory T cells as a new therapeutic target for atherosclerosis. Acta Pharmacol Sin. (2018) 39:1249–58. doi: 10.1038/aps.2017.140 PMC6289392 29323337

[63] Tang Q Adams JY Tooley AJ Bi M Fife BT Serra P. Visualizing regulatory T cell control of autoimmune responses in nonobese diabetic mice. Nat Immunol. (2006) 7:83–92. doi: 10.1038/ni1289 16311599 PMC3057888

[64] Engelbertsen D Andersson L Ljungcrantz I Wigren M Hedblad B Nilsson J. T-helper 2 immunity is associated with reduced risk of myocardial infarction and stroke. Arterioscler Thromb Vasc Biol. (2013) 33:637–44. doi: 10.1161/ATVBAHA.112.300871 23307873

[65] Foks AC Lichtman AH Kuiper J. Treating atherosclerosis with regulatory T cells. Arterioscler Thromb Vasc Biol. (2015) 35:280–7. doi: 10.1161/ATVBAHA.114.303568 PMC4715365 25414253

[66] Vignali DA Collison LW Workman CJ. How regulatory T cells work. Nat Rev Immunol. (2008) 8:523–32. doi: 10.1038/nri2343 PMC2665249 18566595

[67] Kearley J Barker JE Robinson DS Lloyd CM. Resolution of airway inflammation and hyperreactivity after in *vivo* transfer of CD4+CD25+ regulatory T cells is interleukin 10 dependent. J Exp Med. (2005) 202:1539–47. doi: 10.1084/jem.20051166 PMC1350743 16314435

[68] Cope A Le Friec G Cardone J Kemper C. The Th1 life cycle: molecular control of IFN-gamma to IL-10 switching. Trends Immunol. (2011) 32:278–86. doi: 10.1016/j.it.2011.03.010 21531623

[69] Subramanian M Tabas I. Dendritic cells in atherosclerosis. Semin Immunopathol. (2014) 36:93–102. doi: 10.1007/s00281-013-0400-x 24196454 PMC3946524

[70] Fang D Zhu J. Molecular switches for regulating the differentiation of inflammatory and IL-10-producing anti-inflammatory T-helper cells. Cell Mol Life Sci. (2020) 77:289–303. doi: 10.1007/s00018-019-03277-0 31432236 PMC11105075

[71] Dimayuga PC Chyu KY Lio WM Zhao X Yano J Zhou J. Reduced neointima formation after arterial injury in CD4-/- mice is mediated by CD8+CD28hi T cells. J Am Heart Assoc. (2013) 2:e000155. doi: 10.1161/JAHA.113.000155 23702879 PMC3698777

[72] Albany CJ Trevelin SC Giganti G Lombardi G Scottà C. Getting to the heart of the matter: the role of regulatory T-cells (Tregs) in cardiovascular disease (CVD) and atherosclerosis. Front Immunol. (2019) 10:2795. doi: 10.3389/fimmu.2019.02795 31849973 PMC6894511

[73] Fontenot JD Rasmussen JP Gavin MA Rudensky AY. A function for interleukin 2 in Foxp3-expressing regulatory T cells. Nat Immunol. (2005) 6:1142–51. doi: 10.1038/ni1263 16227984

[74] Gottschalk RA Corse E Allison JP. Expression of Helios in peripherally induced Foxp3+ regulatory T cells. J Immunol. (2012) 188:976–80. doi: 10.4049/jimmunol.1102964 22198953

[75] Lord SJ Rajotte RV Korbutt GS Bleackley RC. Granzyme B: a natural born killer. Immunol Rev. (2003) 193:31–8. doi: 10.1034/j.1600-065X.2003.00044.x 12752668

[76] Gravano DM Hoyer KK. Promotion and prevention of autoimmune disease by CD8+ T cells. J Autoimmun. (2013) 45:68–79. doi: 10.1016/j.jaut.2013.06.004 23871638

[77] Simons KH de Vries MR Peters HAB Jukema JW Quax PHA Arens R. CD8+ T cells protect during vein graft disease development. Front Cardiovasc Med. (2019) 6:77. doi: 10.3389/fcvm.2019.00077 31263704 PMC6584838

[78] Steinman RM Witmer MD. Lymphoid dendritic cells are potent stimulators of the primary mixed leukocyte reaction in mice. Proc Natl Acad Sci U.S.A. (1978) 75:5132–6. doi: 10.1073/pnas.75.10.5132 PMC336278 154105

[79] Metlay JP Witmer-Pack MD Agger R Crowley MT Lawless D Steinman RM. The distinct leukocyte integrins of mouse spleen dendritic cells as identified with new hamster monoclonal antibodies. J Exp Med. (1990) 171:1753–71. doi: 10.1084/jem.171.5.1753 PMC2187889 2185332

[80] Gil-Pulido J Zernecke A. Antigen-presenting dendritic cells in atherosclerosis. Eur J Pharmacol. (2017) 816:25–31. doi: 10.1016/j.ejphar.2017.08.016 28822856

[81] Zernecke A. Dendritic cells in atherosclerosis: evidence in mice and humans. Arterioscler Thromb Vasc Biol. (2015) 35:763–70. doi: 10.1161/ATVBAHA.114.303566 25675999

[82] Anzai T. Inflammatory mechanisms of cardiovascular remodeling. Circ J. (2018) 82:629–35. doi: 10.1253/circj.CJ-18-0063 29415911

[83] Mayadas TN Cullere X Lowell CA. The multifaceted functions of neutrophils. Annu Rev Pathol. (2014) 9:181–218. doi: 10.1146/annurev-pathol-020712-164023 24050624 PMC4277181

[84] Kolaczkowska E Kubes P. Neutrophil recruitment and function in health and inflammation. Nat Rev Immunol. (2013) 13:159–75. doi: 10.1038/nri3399 23435331

[85] Amulic B Cazalet C Hayes GL Metzler KD Zychlinsky A. Neutrophil function: from mechanisms to disease. Annu Rev Immunol. (2012) 30:459–89. doi: 10.1146/annurev-immunol-020711-074942 22224774

[86] Mantovani A Cassatella MA Costantini C Jaillon S. Neutrophils in the activation and regulation of innate and adaptive immunity. Nat Rev Immunol. (2011) 11:519–31. doi: 10.1038/nri3024 21785456

[87] Heydarkhan-Hagvall S Chien S Nelander S Li YC Yuan S Lao J. DNA microarray study on gene expression profiles in co-cultured endothelial and smooth muscle cells in response to 4- and 24-h shear stress. Mol Cell Biochem. (2006) 281:1–15. doi: 10.1007/s11010-006-0168-6 16328952

[88] Misra S Fu AA Puggioni A Glockner JF McKusick MA Bjarnason H. Proteomic profiling in early venous stenosis formation in a porcine model of hemodialysis graft. J Vasc Interv Radiol. (2009) 20:241–51. doi: 10.1016/j.jvir.2008.10.004 PMC2679092 19028119

[89] Arbel Y Finkelstein A Halkin A Birati EY Revivo M Zuzut M. Neutrophil/lymphocyte ratio is related to the severity of coronary artery disease and clinical outcome in patients undergoing angiography. Atherosclerosis. (2012) 225:456–60. doi: 10.1016/j.atherosclerosis.2012.09.009 23040448

[90] Turak O Ozcan F Isleyen A Tok D Sokmen E Buyukkaya E. Usefulness of the neutrophil-to-lymphocyte ratio to predict bare-metal stent restenosis. Am J Cardiol. (2012) 110:1405–10. doi: 10.1016/j.amjcard.2012.07.003 22858185

[91] Yilmaz H Bozkurt A Cakmak M Celik HT Bilgic MA Bavbek N. Relationship between late arteriovenous fistula (AVF) stenosis and neutrophil-lymphocyte ratio (NLR) in chronic hemodialysis patients. Ren Fail. (2014) 36:1390–4. doi: 10.3109/0886022X.2014.945183 25246339

[92] Bashar K Zafar A Ahmed K Kheirelseid EA Healy D Clarke-Moloney M. Can a neutrophil-lymphocyte ratio derived from preoperative blood tests predict arteriovenous fistula maturation? Ann Vasc Surg. (2016) 35:60–7. doi: 10.1016/j.avsg.2016.02.020 27263823

[93] Silvestre-Roig C Braster Q Ortega-Gomez A Soehnlein O. Neutrophils as regulators of cardiovascular inflammation. Nat Rev Cardiol. (2020) 17:327–40. doi: 10.1038/s41569-019-0326-7 31996800

[94] Soehnlein O Kai-Larsen Y Frithiof R Sorensen OE Kenne E Scharffetter-Kochanek K. Neutrophil primary granule proteins HBP and HNP1–3 boost bacterial phagocytosis by human and murine macrophages. J Clin Invest. (2008) 118:3491–502. doi: 10.1172/JCI35740 PMC2532980 18787642

[95] Doring Y Drechsler M Soehnlein O Weber C. Neutrophils in atherosclerosis: from mice to man. Arterioscler Thromb Vasc Biol. (2015) 35:288–95. doi: 10.1161/ATVBAHA.114.303564 25147339

[96] Ortega-Gomez A Salvermoser M Rossaint J Pick R Brauner J Lemnitzer P. Cathepsin G controls arterial but not venular myeloid cell recruitment. Circulation. (2016) 134:1176–88. doi: 10.1161/CIRCULATIONAHA.116.024790 PMC5288007 27660294

[97] Kosar F Varol E Ayaz S Kütük E Oğuzhan A Diker E. Plasma leukocyte elastase concentration and coronary artery disease. Angiology. (1998) 49:193–201. doi: 10.1177/000331979804900305 9523542

[98] Dollery CM Owen CA Sukhova GK Krettek A Shapiro SD Libby P. Neutrophil elastase in human atherosclerotic plaques: production by macrophages. Circulation. (2003) 107:2829–36. doi: 10.1161/01.CIR.0000072792.65250.4A 12771009

[99] Wen G An W Chen J Maguire EM Chen Q Yang F. Genetic and pharmacologic inhibition of the neutrophil elastase inhibits experimental atherosclerosis. J Am Heart Assoc. (2018) 7:e008187. doi: 10.1161/JAHA.117.008187 29437605 PMC5850208

[100] Galluzzi L Chan TA Kroemer G Wolchok JD López-Soto A. The hallmarks of successful anticancer immunotherapy. Sci Transl Med. (2018) 10:eaat7807. doi: 10.1126/scitranslmed.aat7807 30232229

[101] He W Xing X Wang X Wu D Mitragotri S. Nanocarrier-Mediated cytosolic delivery of biopharmaceuticals. Advanced Funct Materials. (2020) 30:1910566. doi: 10.1002/adfm.201910566

[102] Goldstein JL Brown MS. A century of cholesterol and coronaries: from plaques to genes to statins. Cell. (2015) 161:161–72. doi: 10.1016/j.cell.2015.01.036 PMC4525717 25815993

[103] Chang HH Chang YK Lu CW Huang CT Chien CT Hung KY. Statins improve long term patency of arteriovenous fistula for hemodialysis. Sci Rep. (2016) 6:22197. doi: 10.1038/srep22197 26902330 PMC4763284

[104] Zhao C Zuckerman ST Cai C Kilari S Singh A Simeon M. Periadventitial delivery of simvastatin-loaded microparticles attenuate venous neointimal hyperplasia associated with arteriovenous fistula. J Am Heart Assoc. (2020) 9:e018418. doi: 10.1161/JAHA.120.018418 33283594 PMC7955373

[105] Cui J Kessinger CW Jhajj HS Grau MS Misra S Libby P. Atorvastatin reduces *in vivo* fibrin deposition and macrophage accumulation, and improves primary patency duration and maturation of murine arteriovenous fistula. J Am Soc Nephrol. (2020) 31:931–45. doi: 10.1681/ASN.2019060612 PMC7217409 32152232

[106] Farokhzad OC Langer R. Impact of nanotechnology on drug delivery. ACS Nano. (2009) 3:16–20. doi: 10.1021/nn900002m 19206243

[107] Misra S Kilari S Yang B Sharma A Wu CC Vazquez-Padron RI. Anti human CX3CR1 VHH molecule attenuates venous neointimal hyperplasia of arteriovenous fistula in mouse model. J Am Soc Nephrol. (2021) 32:1630–48. doi: 10.1681/ASN.2020101458 PMC8425661 33893223

[108] Chapman NM Chi H. mTOR signaling, Tregs and immune modulation. Immunotherapy. (2014) 6:1295–311. doi: 10.2217/imt.14.84 PMC4291176 25524385

[109] Schofer J. Sirolimus-eluting stents for treatment of patients with long atherosclerotic lesions in small coronary arteries: double-blind, randomised controlled trial (E-SIRIUS). Lancet. (2003) 362:1093–9. doi: 10.1016/S0140-6736(03)14462-5 14550694

